# The Effect of Everolimus in an In Vitro Model of Triple Negative Breast Cancer and Osteoclasts

**DOI:** 10.3390/ijms17111827

**Published:** 2016-11-01

**Authors:** Laura Mercatali, Chiara Spadazzi, Giacomo Miserocchi, Chiara Liverani, Alessandro De Vita, Alberto Bongiovanni, Federica Recine, Dino Amadori, Toni Ibrahim

**Affiliations:** Osteoncology and Rare Tumors Center, Istituto Scientifico Romagnolo per lo Studio e la Cura dei Tumori (IRST) IRCCS, 47014 Meldola, Italy; chiara.spadazzi@irst.emr.it (C.S.); giacomo.miserocchi@irst.emr.it (G.M.); chiara.liverani@irst.emr.it (C.L.); alessandro.devita@irst.emr.it (A.D.V.); alberto.bongiovanni@irst.emr.it (A.B.); federica.recine@irst.emr.it (F.R.); dino.amadori@irst.emr.it (D.A.); toni.ibrahim@irst.emr.it (T.I.)

**Keywords:** breast cancer, bone metastasis, everolimus, triple negative

## Abstract

Metastatic bone disease has a major impact on morbidity of breast cancer (BC) patients. Alterations in mTOR signaling are involved both in cancer progression and in osteoclast differentiation. The purpose of this study was to assess the role of mTOR inhibitor Everolimus (Eve) on osteoclastogenesis induced by triple negative BC cells. To this aim, we developed an in vitro human model of osteoclastogenesis from peripheral blood monocytes co-cultured with the triple negative SCP2 and the hormonal receptor positive MCF7 cell lines. Osteoclastogenesis was evaluated by TRAP staining, evaluation of F actin rings and Calcitonin Receptor expression. Eve significantly reduced differentiation induced by cancer cells and resulted more effective when evaluated in combination with Denosumab and Zoledronic Acid (Zol). Combination with Zol showed a total abrogation of osteoclast differentiation induced by the triple negative cell line, not by MCF7. Finally, we observed that Eve was active in the inhibition of the crosstalk between cancer cells and osteoclasts reproduced by our model, highlighting a new therapeutic choice for the subsetting of triple negative BC patients. We observed a difference in the response to bone-targeted therapy with respect to BC subtypes. Our model may represent a valid platform for preclinical trials on bone-targeted drugs and for the study of the interplay of BC with bone stromal cells.

## 1. Introduction

Bone is the most common site of breast cancer (BC) metastasis, with 70% of deceased BC patients carrying evidence of bone metastasis [1]. Bone relapse impairs patient outcome and quality of life, due to pain, pathologic fractures, spinal compression and hypercalcemia [2]. The tumor environment contributes to metastasis formation, in which stromal cells are involved [3,4]. The crosstalk between tumor and stromal cells is crucial for cancer progression both in primary tissue and secondary sites. In particular, osteolytic bone metastasis from BC is the result of a pathological vicious cycle established by the reciprocal interactions between cancer cells (CCs), bone cells and bone microenvironment. CCs break bone homeostasis increasing osteoclastogenesis and, consequently, bone resorption [5,6,7]. Osteoclasts are the bone cells responsible for bone resorption in normal and pathologic conditions. The receptor activator of nuclear factor-κB ligand (RANK-L) binds and activates its receptor RANK on the surface of preosteoclasts stimulating their differentiation and maturation, leading to an increase in bone resorption. Osteoprotegerin (OPG) inhibits osteoclastogenesis, binding to RANKL as a decoy receptor [8]. BC cells secrete soluble mediators that drive osteoclastogenesis directly, by enhancing osteoclast differentiation and activity, and by indirectly stimulating osteoblasts to increase RANKL expression, with an increase in RANKL/OPG ratio and a following increase in osteoclastogenesis. The unbalanced activation of osteoclasts results in a massive bone resorption, which, in turn, causes the release of the growth factors (GFs) stored in the bone matrix, promoting tumor growth with the establishment of a self-maintained vicious cycle [9].

The mTOR pathway is an important regulator of cell signaling with known roles in physiological processes (including cell growth, survival and autophagy) and in cancer [10]. In recent years, some published results reported that mTOR is involved also in bone diseases. mTOR is implicated in osteoclastogenesis: in particular, M-CSF, RANKL and TNF-α have shown to exert their anti-apoptotic effects on osteoclasts through mTOR/S6K. mTOR inhibition has already been proven a successful strategy for the control of tumor growth in different types of cancers [11,12]. Emerging evidence suggests that the mTOR pathway may play a role in the development and progression of bone cancer. The combination of mTOR inhibitor Everolimus (Eve) with the aromatase inhibitor Exemestane was observed to significantly increase progression free survival of BC patients treated with Exemestane as a single agent with hormonal receptor positive tumors (Bolero Trial) [13,14]. Moreover, bone turnover was significantly inhibited in patients treated with Eve plus Exemestane. It is thus assumed that the mechanism of action of the drug has an inhibitory effect on bone turnover coupled with an anti-tumor activity. mTOR inhibition could impact bone metastasis progression, contributing to both metastasis progression inhibition and bone health maintenance, improving survival and quality of life in BC patients treated with aromatase inhibitors [15]. Although strong clinical evidence of the role of Eve in the treatment of triple negative BC is still lacking, some recent studies have suggested investigating its impact also in this setting of patients. A phase-two study reported a clinical benefit rate of 36% from the use of Carboplatinum and Eve. Furthermore, mesenchymal-like triple negative BC subtype was found to be sensitive to dual PI3K/mTOR inhibition in preclinical models [16,17].

In order to highlight the benefits of Eve in the treatment of bone metastasis of triple negative BC patients, we assessed it on an improved preclinical model of bone metastasis previously developed [18]. Human monocytes taken from peripheral blood were induced to differentiate into osteoclasts by GFs directly or indirectly by the osteotropic triple negative SCP2 cell line [19]. We analyzed the effects of Eve as a single agent and in combination with the standard bone-targeted therapies Zoledronic acid (Zol) and Denosumab (Den) in the inhibition of osteoclastogenesis. Our model enabled the study of the drug mechanism of action in breaking the interaction between cancer and stromal cells. We also compared the results obtained from the inhibition of osteoclastogenesis induced by SCP2 with those of osteoclastogenesis induced by MCF7, in order to understand the potential efficacy of Eve treatment in metastatic triple negative BC patients.

## 2. Results

### 2.1. Osteoclastogenesis Model Induced by SCP2

#### 2.1.1. Indirect Co-Cultures

**Figure 1:**
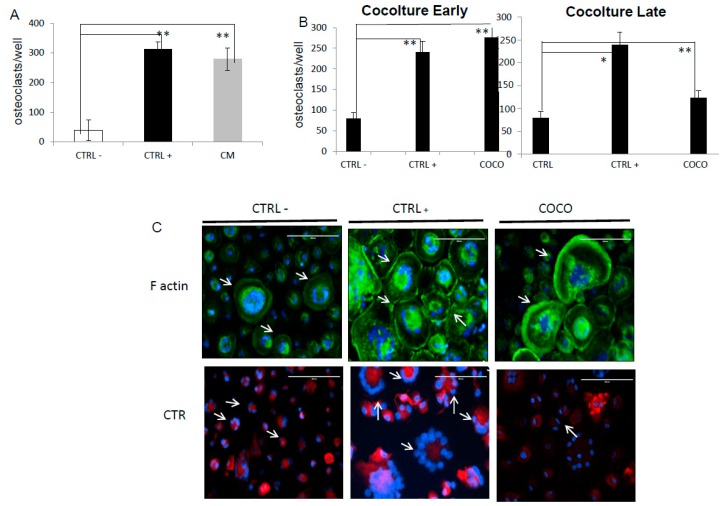
Co-culture optimization: (**A**) Indirect co-cocolture Conditioned medium (CM) collected by SCP2 cultures was added to complete α-MEM to obtain a CM with 20% cancer cell medium and 80% α-MEM. SCP2 CM sustained osteoclastogenesis statistically significantly compared to CTRL−. Significance to Bonferroni test (performed after Anova): * *p* < 0.05;** *p* < 0.001; (**B**) DIRECT COCO: Direct COCO were obtained seeding cancer cells (CCs) on trasnwell inserts on 24 well plates in which PBMCs were seeded; in this way, crosstalk between cells was allowed by medium sharing. (**B**) Direct co-coltures: We evaluated whether the effect of CCs on osteoclastogenesis was different following early (Days 1–7) or late (Days 7–13) interaction between CCs and PBMCs in the differentiation period. Anova *p* value was significant when CTRL−, CTRL+ and early COCO were analyzed; Bonferroni test showed the following comparisons as statistically significant: CTRL− vs. CTRl+: *p* = 0.009; CTRL− vs. early COCO: *p* = 0.003; Anova *p* value was significant when CTRL−, CTRL+ and late COCO were analyzed; Bonferroni test showed the following comparisons as statistically significant: CTRL− vs. CTRL+: *p* = 0.008; CTRL+ vs. late COCO: *p* = 0.047. Significance to Bonferroni test: * *p* < 0.05, ** *p* < 0.001; (**C**) Phalloidin staining (**green**) to detect F actin rings and calcitonin receptor (CTR) expression (**red**) on osteoclasts in all conditions and indicated by the arrows. The scale bar is 100 µm.

To understand whether SCP2 conditioned medium effectively induces osteoclast differentiation, an osteoclastogenesis assay was performed starting from human peripheral blood mononuclear cells (PBMCs). Conditioned media from SCP2 (CM) and RANKL-MCSF-supplemented media (differentiation media used as positive control CTRL+) significantly induced osteoclastogenesis in. The mean number of OC cell-like cells (with >4 nuclei and positive to TRAP staining, as described in the experimental section) was 313 ± 42 and 279 ± 76 when differentiated by differentiaiton and conditioned media (CM), respectively) (Figure 1A). As Anova analysis was statistically significant, we performed Bonferroni test showing statistical difference between CTRL+ and CTRL− (*p* = 0.003), and between CM and CTRL− (*p* = 0.001).

#### 2.1.2. SCP2-PBMCs Direct Co-Culture (COCO)

We developed a direct COCO system better representative of the interactions between CCs and PBMCs with respect to indirect COCO thanks to their mutual influence. We analyzed the CC role in osteoclastogenesis at an early phase of the assay, from Day 1 to Day 7 of differentiation, and at a later phase, from Day 7 to Day 13, to determine in which phase CC soluble mediators contributed the most. The number of OC cell-like cells cultured with CCs in the early COCO (216 ± 32) doubled compared to those obtained in the late COCO (123 ± 27). The contribution of CCs was similar (Figure 1B) to CTRL+ when COCO was performed early; meanwhile, their effect was significantly lower than CTRL+ (*p* = 0.047) when COCO was performed later. As human PBMCs culture could include activated macrophage polycarions expressing TRAP besides osteoclasts [20], we assessed the presence of F actin rings and the expression of calcitonin receptor (CTR)—Two hallmarks of osteoclasts to ensure that TRAP+ cells obtained at the end of the assay were osteoclasts (Figure 1C) [21]. We reported positivity to both markers of cells seeded in all the analyzed conditions, confirming the presence of the osteoclasts in all conditions, in particular in the CTRL−, due to the spontaneous osteoclastogenesis that can occur even in absence of as previously reported [18,22].

#### 2.1.3. Soluble Mediator Profile during Osteoclastogenesis

The presence of markers involved in osteoclastogenesis and bone metastasis development [18,23,24] as ICAM1, RANKL, MCSF1, and IL-6 was evaluated in culture media of osteoclasts and SCP2 CCs. In particular, soluble mediator concentrations were evaluated at baseline (Day 3 of culture), on Day 6 of culture and after Eve administration. RANKL was detected in none of the samples; ICAM1 was detected at very low levels.

**Figure 2:**
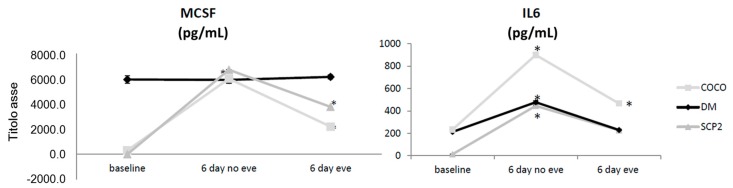
Cytokine secretion over time and after Eve treatment: soluble mediators over time and in presence/absence of Eve. T test was performed; Significance to *t* test: * *p* < 0.05.

MCSF baseline levels in COCO were about 20-fold lower than in differentiation media (DM) condition, but reached similar levels after six days; MCSF levels in SCP2 after six days of cultures showed the same trend as COCO. IL-6 levels were lower also at baseline in SCP2 than in other conditions. After six days of culture, levels in all conditions increased reaching statistical significance (*p* = 0.01 for SCP2, *p* = 0.04 for DM, and *p* = 0.005 for COCO) (Figure 2).

#### 2.1.4. Eve Effect on Early and Late Osteoclastogenesis in an Indirect Co-Culture Model System

**Figure 3:**
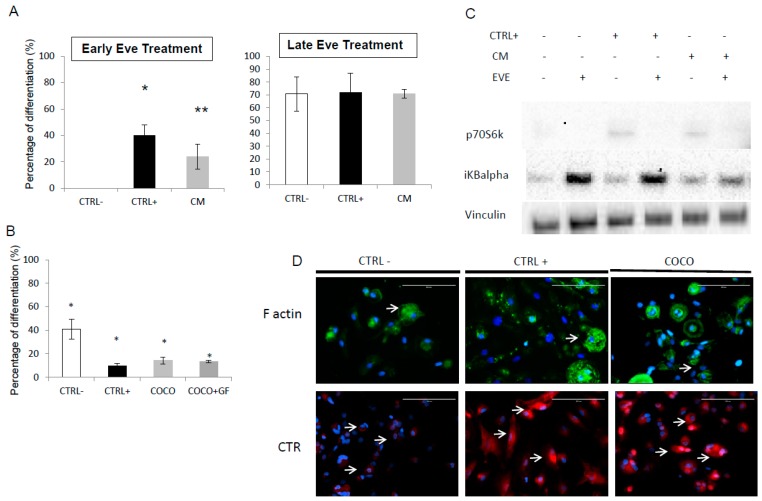
Everolimus effect on osteoclastogenesis treatment: Eve effect on osteoclastogenesis treatment: (**A**) INDIRECT COCO: percentage of differentiation with respect to untreated samples after administration on Day 3 of osteoclastogenesis assay (early) and on Day 10 (late). The experiment was performed as described for Figure 1. Significance: * *p* < 0.05; ** *p* < 0.01 Eve was used at a concentration of 0.1 μg/mL; (**B**) DIRECT COCO: percentage of differentiation after treatment with Eve with respect to untreated samples. The significance is related to the TRAP + polykarions number change in treated vs. untreated samples in the same conditions. Significance: * *p* < 0.01; (**C**) Western blot analysis on osteoclasts treated with Eve for 6 h and conditioned with SCP2 CM or frowth factors (CTRL+) IKBalpha and p70S6K were evaluated. The latter protein was expressed only in presence of GF or CM and was downregolated after Eve administration; (**D**) Phalloidin and CTR staining to detect F actin ring and CTR expression on osteoclasts in all different conditions treated with Eve. The scale bar is 100 µm.

As osteoclastogenesis assay has a duration of 14 days, Eve effect was evaluated on Day 3 (early Eve treatment) and on Day 10 of treatment (late Eve treatment) to identify the time point in the differentiation process when the drug has a greater effect on the inhibition of osteoclast maturation. Both CM and CTRL+ induced TRAP+ cells were significantly sensitive to the drug. After early Eve treatment no OC cell-like cells were observed in the CTRL− compared to 41 ± 16 cells observed after late Eve treatment. With respect to the other conditions, OC cell-like cells decreased from 313 ± 41 to 95 ± 19 (*p* = 0.0002) in CM and from 279 ± 75 to 57 ± 22 in DM (*p* = 0.01). Eve effect was higher if administered early, with 20%–30% differentiation in the treated samples compared to their respective controls. Late Eve treatment, instead, produced a decrease in differentiation by about 25% (Figure 3A). As Eve resulted more effective in early administration, the following experiments were performed with the same schedule of treatment.

#### 2.1.5. Eve Effect on Osteoclastogenesis in the Direct COCO System

On the basis of the results discussed above, SCP2 influence was more effective on osteoclast differentiation in early Eve treatment: in the following experiments, cell COCOs were started on Day 1 of the osteoclastogenesis assay. Eve treatment was less effective in samples conditioned with CCs (with/without GF) than in the CTRL+ condition without reaching statistical significance; (Figure 3B). In particular, the drug produced 60%–90% osteoclastogenesis inhibition; the differentiation decrease was statistically significant in all the tested conditions. We analyzed by Western blot the changes after treatment of p70S6k, a protein activated downstream mTOR signaling. We observed a decrease in p70S6k levels in samples treated with Eve, confirming the block of the mTOR pathway (Figure 3C). Polycarion cells of all conditions showed a loss of F actin ring distribution after Eve administration. CTR expression was not lost due to Eve administration.

#### 2.1.6. Inhibition of Osteoclastogenesis Induced by Bone-Targeted Therapy

Eve was observed to be active in the first part of the osteoclastogenesis assay. Den is known to inhibit the RANKL binding on preosteoclasts in the second part of the differentiation process. In order to evaluate the activity of the combination of the two drugs, cells were treated with Eve on Day 3 for 72 h, then with Den on Day 7 for 72 h after a 24-h washout. Zol treatment was performed with the same schedule as Den (see the Experimental section for schedule details).

**Figure 4:**
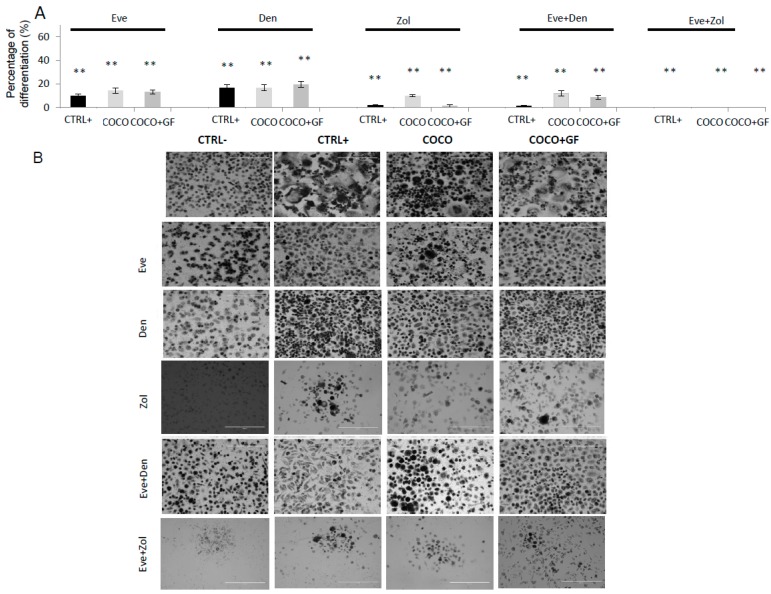
Eve treatment on osteoclastogenesis induced by SCP2 as a single agent and in combination: Drug effect on osteoclastogenesis Eve was used at a concentration of 0.1 μg/mL. (**A**) Percentage of differentiation with respect to untreated samples after Eve and other bone-targeted drugs administration; Anova analysis and Bonferroni Test were performed when appropriate; for all conditions (CTRL+, COCO; COCO + GF) a significant decrease in OC cell-like cells was observed in treated samples with respect to those untreated (** *p* < 0.00001); (**B**) Pictures of all experimental conditions performed at 10× magnification.

Den as single agent showed about 80% inhibition of osteoclastogenesis in all the tested conditions (Figure 4A). Bonferroni Test showed no statistically significant difference between Den and Eve as single agent. With respect to the TRAP+ polykarion number, the drug combination resulted in osteoclastogenesis inhibition of 89% and 92% in COCO + GF and COCO, respectively. On the other hand, the DM-conditioned cells were almost totally inhibited (98%) (Figure 4A). Zol treatment was more effective on osteoclast differentiation than Den. The OC cell-like cell number in CTRL+ and in COCO + GF conditioned were reduced to about 1.5% The combination of drugs resulted in a total inhibition of osteoclastogenesis in all samples without reaching statistical significance with respect to drug administered solely.

#### 2.1.7. Drug Effect on CCs

**Figure 5:**
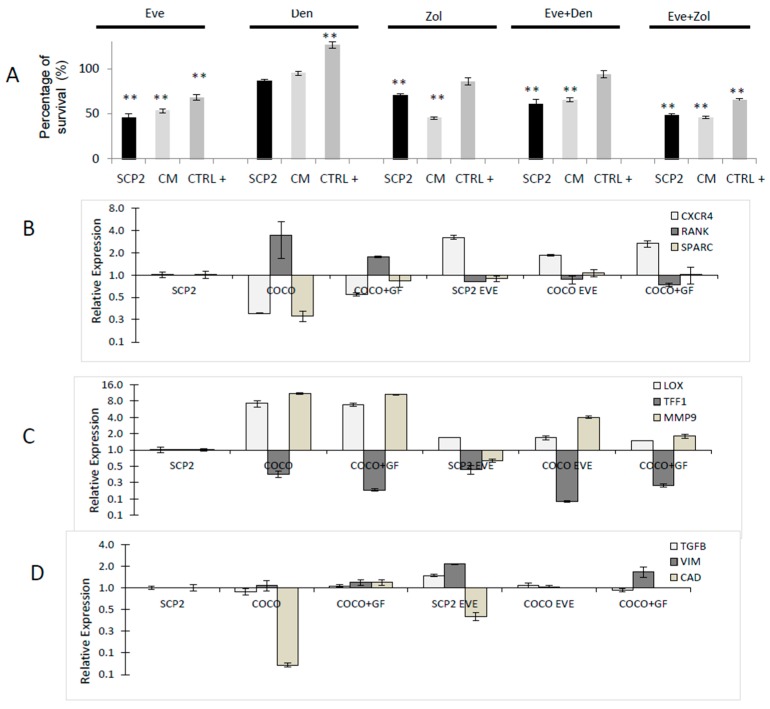
Drug effect on SCP2 CCs. In this case, SCP2 were indirectly co-cultured with osteoclast CM or DM as described in the Experimental section. Eve was used at a concentration of 0.1 μg/mL, Den at a concentration of 5 μg/mL, Zol at a concentration of 10 μM. (**A**) MTT analysis on cancer cells. As Anova Analysis resulted significant in all conditions (SC P2, CM, DM), Bonferroni Test was performed; in all conditions survival had significantly decreased compared to CTRL−. (** *p* < 0.001). Gene expression analyses on CCs cultured solely and with PBMCs. Markers of osteomimicry (**B**); CC aggressiveness (**C**); and EMT (**D**) were analyzed in presence/absence of Eve. Data were normalized with respect to untreated SCP2 cultured solely and graphed on a double logarithmic scale.

The effect of Eve and bone-targeted therapy was evaluated on SCP2 indirectly conditioned with osteoclast CM or GFs MCSF and RANKL (CTRL+ obtained with DM) (Figure 5A).

Den was the less effective drug in lowering CC survival. Eve proved the most effective drug on SCP2 cultured solely showing 45% survival, compared to 86% and 70% obtained with Den and Zol, respectively (Figure 5A). Osteoclast CM seemed to protect CCs from Eve effect as CCs cultured with osteoclast CM or DM were less sensitive to Eve. The same trend was observed also for Den; in this case, the slight decrease in survival compared to the CTRL in SCP2 cultured solely was completely lost in CM and DM samples. Survival after Zol was 45% in CM, whereas 70% and 85% in SCP2 cultured alone and with GFs, respectively. The combination of Eve with either Den or Zol showed no improvement in the effect of Eve (Figure 5A).

**Figure A1:**
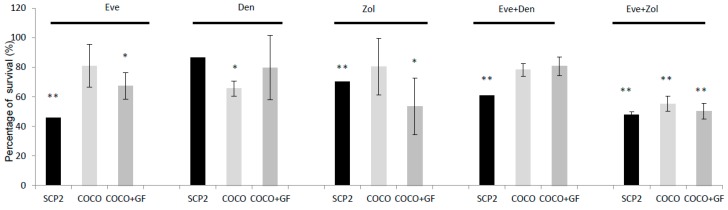
Drug effect on SCP2 directly co-cultured with osteoclasts; significance related to the corresponding untreated sample: * *p* < 0.05 and ** *p* < 0.001.

We repeated MTT analysis with SCP2 co-cultured directly with towards OC differentiation, obtaining comparable results (Figure A1).

#### 2.1.8. Drug Effects on CC Gene Expression

Gene expression analysis was performed on CCs in presence/absence of monocytes/preosteoclasts to evaluate the modulation of some markers of osteomimicry, EMT and aggressiveness (Figure 5B–D).

Osteoclasts induced SCP2 to express RANK, LOX and MMP9; Cad and TFF1 expression, instead, decreased when cells interacted with monocytes/preosteoclasts. CCs surviving Eve treatment experienced a decrease in RANKexpression and an increase in CXCR4 in all the analyzed samples compared to their respective CTRL. We also observed the upregulation of VIM and the downregulation of CAD expression, showing a switch towards mesenchymality.

#### 2.1.9. Drug Effects on Soluble Mediator Profile

MCSF levels were not affected in DM, while lowered significantly after Eve in COCO and SCP2 samples compared to the untreated samples (*p* = 0.03 and *p* = 0.0002, respectively). IL-6 modulation decreased in all samples after treatment with Eve (Figure 2).

### 2.2. Osteoclastogenesis Model Induced by MCF7

#### 2.2.1. Osteoclast Direct COCO

**Figure 6:**
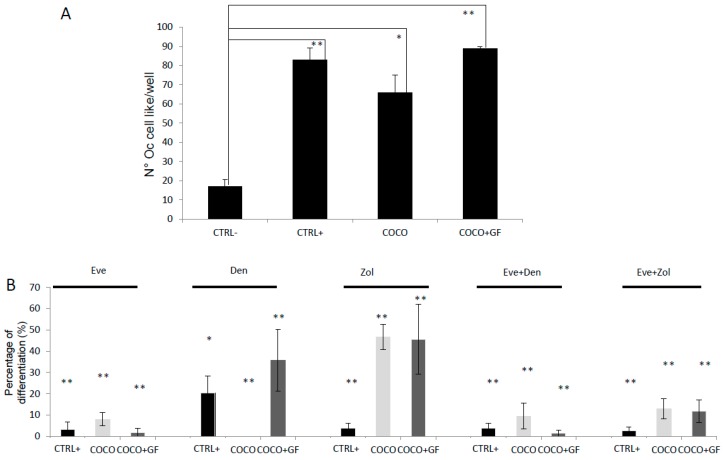
Osteoclastogenesis induced by MCF7 and response to drugs. (**A**) Osteoclastogenesis assay with direct COCO of MCF7; we performed Bonferroni Test obtaining significance in the following comparisons: CTRL− vs. DM: *p* = 0.007; CTRL− vs. COCO: *p* = 0.027; CTRL− COCO + GF: *p* = 0.007; (**B**) Drug effect on osteoclastogenesis; percentage of differentiation with respect to untreated samples after Eve and other bone-targeted drugs administration; Anova analysis and Bonferroni Test were performed when appropriate; in all conditions (CTRL+, COCO; COCO + GF) a significant decrease in OC cell-like cells was observed in treated samples compared to those untreated with * *p* < 0.05 and ** *p* < 0.01.

MCF7 were able to significantly induce differentiation of osteoclasts either in presence or in absence of GFs (Figure 6A).

#### 2.2.2. Drugs Effect on Osteoclastogenesis Induced by MCF7

Eve significantly inhibited the osteoclastogenesis induced by MCF7 in absence and in presence of GFs by about 92% and 98%, respectively (both *p* = 0.0001). Den totally abrogated osteoclastogenesis in monocytes differentiated by MCF7 and reduced the differentiation by about 30% when GFs were added to the wells (both *p* = 0.0001). Zol was less effective than Den in COCO and COCO + GF samples as reported in Figure 6B.

The combination of Eve plus Den or Zol increased osteoclastogenesis inhibition not significantly. We compared the drug effect on the osteoclasteogenesis induced by SCP2 or MCF7. Eve produced a stronger effect on osteoclasts induced by MCF7. Den resulted more effective on the inhibition of osteoclastogenesis induced by MCF7, whereas Zol had stronger effects on osteoclast differentiation induced by SCP2.

#### 2.2.3. Drug Effects on CC Survival

**Figure 7:**
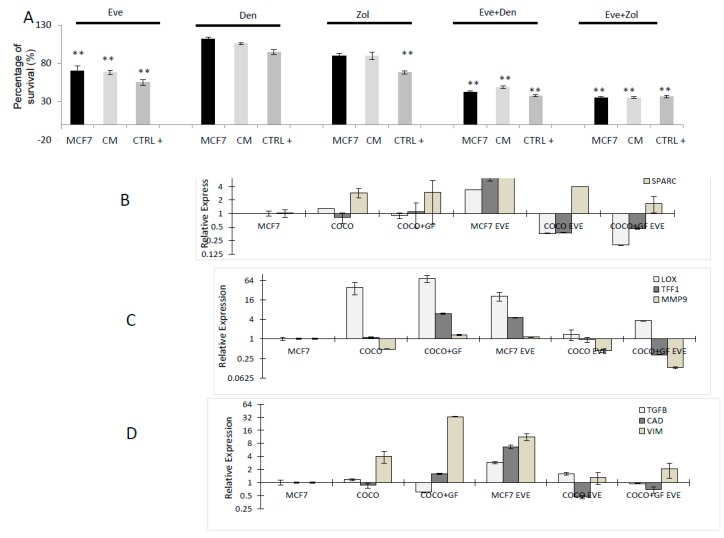
Drug effect on MCF7 CCs. (**A**) MTT analysis on CCs. As Anova analysis of drug effects resulted significant in all conditions (SCP2, CM, DM), Bonferroni Test was performed; in all conditions all drugs significantly decreased survival compared to CTRL− (significance related to the corresponding untreated sample: * *p* < 0.05 and ** *p* < 0.001). (**B**) Gene expression analysis on CCs cultured solely and with osteoclasts. Markers of osteomimicry (**B**); CC aggressiveness (**C**); and EMT (**D**) were analyzed in presence/absence of Eve. Data were normalized with respect to untreated MCF7 cultured singularly and graphed on a double logarithmic scale.

MCF7 cells in our experimental conditions were less sensitive to Eve than to the triple negative cell line with 55%–70% survival in the different conditioned samples. Response to Den was similar to SCP2, whereas Zol treatment was less effective in MCF7. Both combinations significantly decreased the percentage of surviving cells compared to drugs used as single agents with *p* ≤ 0.01 in all conditions (SCP2, DM and CM) (Figure 7A).

#### 2.2.4. Drug Effects on CC Gene Expression

MCF7 expressed higher levels of SPARC and LOX and VIM when they interacted with osteoclasts. MCF7 cells cultured alone and surviving Eve treatment showed an increased expression in the osteomimicry markers CXCR4, RANK and SPARC and LOX, in MCF7 cultured alone. CCs cultured with osteoclasts were observed to reduce TFF1 and MMP9 levels after treatment (Figure 7B).

## 3. Discussion

The primary aim of this study was to evaluate Eve treatment as a possible strategy to interrupt the interactions established between BC and stromal cells in a preclinical model of bone metastasis. The role of Eve in the treatment of patients with bone metastasis from triple negative BC has not yet been fully elucidated. We used an optimized fully human preclinical model mimicking bone metastasis to investigate the efficacy of Eve as a single agent or in combination with conventional bone-targeted therapy to inhibit osteoclastogenesis in this setting of aggressive, non-responsive cancer. Eve seemed a suitable candidate for the treatment of bone metastasis since the mTOR pathway plays a key role both in CCs and in osteoclast proliferation and survival. We previously developed an in vitro model of bone metastasis that we further improved; human monocytes were induced to differentiate into osteoclasts by direct interaction with triple negative osteotropic BC cell line SCP2. After isolation from the parental MDA-MB-231 for its high tropism to the bone in a mouse model [19], the in vitro bone metastasis model presented the same molecular phenotypic features as bone metastatic BC cells, which can recapitulate the molecular interactions among cells in the bone microenvironment.

Hussein et al. observed that Rapamycin, an analogous of Eve, significantly decreased the osteoclast population and osteolysis associated with experimental metastases in an in vivo model of bone metastasis developed by inoculating the murine triple negative BC cell line 4T1 [25]. Their observations are consistent with our results. Our data indicated that Eve significantly inhibits osteoclastogenesis. In particular, we observed that Eve was more effective when administered early in the osteoclast differentiation. We thus assumed that the mTOR pathway was involved in the first phase of the differentiation process. This hypothesis was supported by a study reporting that the mTOR signaling activation by M-CSF is a requisite for differentiation [26,27]. As a confirmation, we observed that in COCO condition, after Eve treatment, MCSF levels decreased by about 50% with respect to the untreated CTRL. We also analyzed osteoclastogenesis induced by directly co-culturing monocytes with the hormonal receptor positive cell line MCF7, which was shown to support osteoclastogenesis as a DM [28]. Our results demonstrated that Eve had greater inhibitory effects on osteoclastogenesis when differentiation was induced by MCF7 as reported by Simone et al. [28]. We reported the effect of the different drugs also on CC survival. SCP2 were more sensitive to Eve than MCF7. Moreover, we observed that Zol as a single agent was more effective on the inhibition of osteoclastogenesis induced by the triple negative cell line than MCF7, with similar results to those obtained for CC survival. Osteoclastogenesis was totally abrogated with the combination of Zol plus Eve.

These results suggested that Zol plus Eve could be useful in the treatment of patients with bone metastasis from triple negative BC. The results are supported by our previous data on the effect of Zol on MCF7 and the triple negative BC cell line MDA-MB-231, which highlighted a higher sensitivity of triple negative CCs to Zol [29,30]. Results from a clinical trial on the role of Zol in the treatment of BC patients in neoadjuvant setting reported a higher percentage of complete response when Zol was added to chemotherapy in triple negative BC patients than in patients with luminal cancers (35% vs. 9.9%) [31]. Supported by clinical observations, the obtained in vitro data confirmed that our preclinical model of bone metastasis is a useful tool for studying drug effects on the bone tumor microenvironment and determining new effective therapeutic strategies.

Denosumab, the antibody anti-RANKL shown to be superior to Zoledronic acid in the prevention of SREs [32], resulted more effective in the blockage of osteoclastogenesis induced by MCF7 than SCP2 in our experimental conditions.

## 4. Materials and Methods

### 4.1. Cell Cultures

Cancer cells: The experiments were performed on SCP2, an osteotropic cell line originating from the triple negative human BC cell line, MDA-MB-231, obtained from the America Type Culture Collection (Rockville, MD, USA) and on the hormonal receptor positive MCF7 (ATCC). SCP2 were kindly provided by Yibin Kang’s laboratory, where they were originally isolated [19]. Cells were cultured as a monolayer in 75-cm^2^ flasks at 37 °C in α-MEM medium (PAA, Piscataway, NJ, USA) supplemented with 1% glutamine (PAA) and 10% fetal bovine serum in a 5% CO_2_ atmosphere. The CM for osteoclastogenic assays was collected by CCs cultured up to 90%–100% confluence, and then supplied with fresh media collected 24 h later, centrifuged, aliquoted and stored at −20 °C.

### 4.2. Osteoclastogenesis Assay

**Figure 8:**
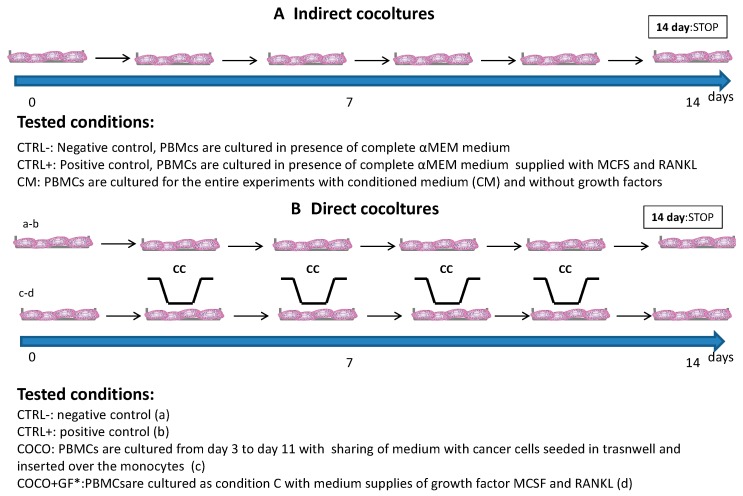
Indirect and direct COCOs: schedules and conditions. Schedule and tested samples of osteoclastogenesis assays are here summarized. PBMCs were differentiated in presence/absence of GFs (CTRL+ and CTRL−, respectively). The influence of CCs on osteoclastogenesis and on drug effects were evaluated with COCOs of CCs and PBMCs in differentiation. We performed indirect COCO (**A**) to optimize the methodology, followed by COCO (**B**) to study the effect of CCs secreted factors on PBMCs and vice versa. * The condition COCO + GF (d) was not tested in all experiments.

Human osteoclasts were obtained from the differentiation of PBMC of healthy donors who gave written informed consent to take part in the study [18]. Monocytes were isolated from buffy coats by Ficoll density gradient as previously described. Briefly, EDTA whole blood (50 mL) was diluted 1:1 with phosphate-buffered saline, layered on lymphocyte separation media (Lymphosep, Biowest, Nuaillé, France) and centrifuged without brakes at 1000× *g* for 20 min. The PBMC layer was collected and washed twice with phosphate-buffered saline (PBS) and treated for 4 min on ice with ACK solution. After having washed them twice with phosphate-buffered, saline cells were resuspended in complete α-MEM (LONZA, Basel, Switzerland). Cells were counted and plated at a concentration of 1,500,000 PBMCs per 2 cm^2^ well. After 24 h, the media was removed and differentiation into osteoclasts was induced by α-MEM supplemented with 20 ng/mL of MCSF (Peprotech, Rocky Hill, NJ, USA) (DM): from Day 8 of culture, RANKL (20 ng/mL) was added with MCSF. The media was changed every 2–3 days and TRAP+ polycarion cells were observed after 14 days of differentiation. To assess CC effects on osteoclastogenesis, both indirect and direct COCOs were performed. Each condition was performed in quadruplicate and experiments were run three times. Figure 8 shows details on schedules and tested conditions.

Indirect COCO: Differentiation into osteoclasts was induced by α-MEM supplemented with 20% SCP2 culture medium (CM) and with 20 ng/mL RANKL and MCSF (CTRL+, obtained supplying cells with DM).

Direct COCO: For COCO assays, 1,500,000 PBMCs per 2 cm^2^ well and, separately, 4000 SCP2 cells were plated in 6.5 mm diameter, 0.4 µm transwell inserts (Corning Ltd., London, UK). Cells were then allowed to adhere to their supports and after 24 h SCP2 (or MCF7)-seeded inserts were placed over the mononuclear cultures to start COCOs. Cells were maintained in COCO for 10 days; PBMCs were left to differentiate until Day 14 of COCO; CCs were detached from transwell and then stored for RNA extraction and gene expression analysis. PBMCs and SCP2 cultured solely were used as CTRL−. COCOs were performed in absence (COCO) and presence of GFs (COCO + GF).

### 4.3. Osteoclast Quantification

The PBMCs left to differentiate, after 14 days were fixed by incubation in 3.7% PBS buffered formaldehyde (Polyscience, Niles, IL, USA) for 20 min at room temperature and then stained for tartrate resistant acid phosphatase (TRAP kit, Sigma-Aldrich, Steinheim, Germany). Nuclei were counterstained with hematoxylin (TRAP kit). Cells with >4 nuclei and positive to TRAP staining were defined OC cells-like cells. Images of differentiated cells were acquired at different magnifications with Axiovision software (Zeiss, Oberkochen, Germany).

### 4.4. Phalloidin and CTR Staining

To detect F actin ring in differentiated cells at the end of the osteoclastogenesis assay described previously, we performed a phalloidin staining as follows. Cells were washed 3 times with PBS and fixed with 4% PFA for 20 min at room temperature followed by permeabilization with 0.1% Triton X-100 for 5 min. The cells were subjected to immunofluorescence staining with phalloidin stock solution (1:40 in PBS) (Life Technologies, Foster City, CA, USA) for 20 min in dark conditions at room temperature. In order to evaluate CTR expression, cells were incubated after fixation with paraformaldehyde (PFA) with an antibody anti CTR (1:40 in 5% Bovine Serum Albumin (BSA)) (Acris Antibodyes, Herford, Germany). Cells were washed 3 times with PBS and incubated for 90 min with a secondary antibody (1/150) conjugated with RFP (Life Technologies) in dark conditions. For both stainings, cells were then washed 3 times with PBS and counterstained with DAPI. Cells were then mounted in ProLong gold and examined by fluorescence microscopy.

### 4.5. Drugs

Eve (Afinitor^®^) and Zoledronic Acid (Zometa^®^) kindly provided by Novartis (East Hanover, NJ, USA), were solubilized at a concentration of 50 mM in sterile water, filtered and stored at −20 °C. Denosumab 120 mg in 1.7 mL (XGEVA^®^) (Thousand Oaks, CA, USA) was stored at 4 °C. Drugs were diluted in complete α-MEM containing 10% FBS, 1% penicillin and 1% l-glutamine.

### 4.6. Drug Exposure and Dose Selection

Doses of Eve, Zol and Den were selected on the basis of plasma levels from pharmacokinetic clinical data [30,33,34]. We used 0.1 μg/mL concentration for Eve, and 5 μg/mL concentration for Den. As Zoledronic acid was reported to accumulate in bone tissues at a higher concentration than plasmatic peak, it was administered to cancer and bone cells at a concentration of 10 μM [30]. Treatment schedule is described in Figure 1. After two days of COCO, cells were exposed to Eve; the drug effect was evaluated in terms of inhibition of osteoclast differentiation at Day 14, and inhibition of CC survival at Day 11 of COCO.

**Figure 9:**
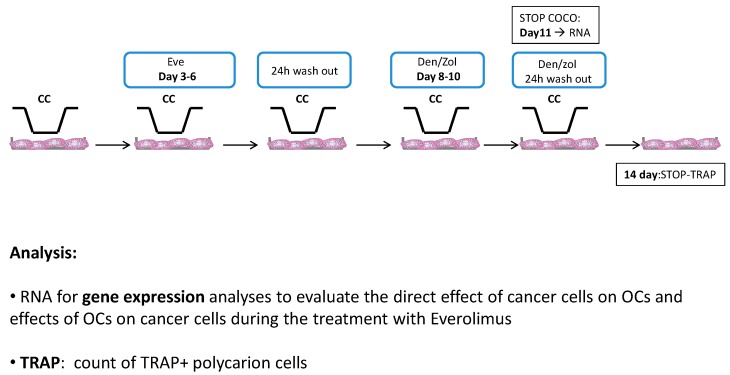
Drug treatment in direct COCO: schedule of Eve administered solely and in combination.

The combination of Eve plus Den/Zol was performed as follows: Eve was administered for 72 h from Day 3 of osteoclastogenesis assay; after a 24-h washout the drug was treated with RANKL. Den/Zol was added to the medium; after additional 72 h, the COCO was terminated; SCP2/MCF7 detached and PBMCs were left to differentiate until Day 14 (Figure 9).

### 4.7. Inhibition of Osteoclastogenesis

To determine osteoclastogenesis inhibition and survival after each drug treatment, the differentiated PBMCs were stained for TRAP as previously described, and counted. The number of OC cell-like cells in treated wells was normalized to untreated wells. Each experiment was performed in quadruplicate and repeated at least 3 times in biological replicates.

### 4.8. Drug Effects Evaluation on CCs

MCF7 and SCP2 cells were seeded at a concentration of 4000 cells per 96-well, and treated with drugs as reported for osteoclastogenesis assays. To evaluate the osteoclast effect on drug efficacy, cells were indirectly conditioned with/without osteoclast CM and GFs. Percentages of survival were then assessed by MMT assay (Sigma Aldrich) according to the manufacturer’s instructions. MTT was performed also on SCP2 directly co-cultured with osteoclasts.

### 4.9. Gene Expression Analyses

Total RNA from CCs and PBMCs differentiated cells cultured solely or in co-colture was isolated using TRIzol Reagent (Invitrogen, Carlsbad, CA, USA) following the manufacturer’s instructions. Five hundred nanograms RNA were reverse-transcribed using the iScript cDNA Synthesis Kit (BioRad, Hercules, CA, USA). Real-Time PCR was performed on the 7500 Real-Time PCR System (Applied Biosystems, Foster City, CA, USA) using TaqMan gene expression assay mix (Applied Biosystems). The stably expressed endogenous β-actin and HPRT genes were used as reference. The following markers were analyzed in cancer cells: CXCR-4, RANK SPARC, MMP-9, HPSE and TGF-β, TFF1, Vimentin, and cadherin (Life Technologies).

### 4.10. MCSF, RANKL, IL-6 and ICAM1 Evaluation

MSCF, ICAM1, IL-6 and RANKL secretion were evaluated in SCP2 and osteoclast media by ELISA kit (R&D systems, Minneapolis, MN, USA for MCSF, IL-6 and ICAM1, Immunodiagnostik AG, Bensheim, Germany, for RANKL) according to the manufacturer’s instructions.

### 4.11. Western Blot

Proteins were lysed by a lysis buffer composed of 50 mM Tris–HCl (pH 8), 150 mM NaCl, 1% Triton X-100 and 0.1% Sodium Dodecyl Sulphate (SDS), supplemented with 1 mM phenylmethylsulfonyl fluoride, and 1:100 protease inhibitors (Sigma-Aldrich). The protein content was quantified using the BCA protein assay kit (Thermo Fisher Scientific, Waltman, MA, USA). An equal amount of protein from each sample was separated on Criterion™ Precast Gel Tris–HCl (Biorad, Hercules, CA, USA) and transferred to polyvinylidene fluoride membranes (Millipore Corporation, Billerica, MA, USA). The membranes were blocked for 1 h in 5% non-fat dry milk PBS with 0.1% Tween 20 (Sigma-Aldrich) at room temperature and incubated with primary antibody overnight at 4 °C. After washing, the membranes were incubated for 1 h at room temperature with horseradishperoxidase-conjugated secondary antibody. The following primary antibodies were used: anti pk70S6 kinase (1:1000 Cell Signalling Technology, Beverly, MA, USA), anti IκB-α (1:1000) (Cell Signalling Technology) anti-Vinculin (1:1000) (Thermo Fisher Scientific). For osteoclast evaluation, cell lysates were collected on Day 3 from osteoclasts stimulated in CM or DM (SCP2) or from CTRL, after a 6-h treatment with Eve.

### 4.12. Statistical Analysis

Each experiment was repeated at least three times and with 4 technical replicates for each condition. Data are presented as mean ± SD. Student’s *t*-test or one way analysis of variance (ANOVA) followed by Bonferroni’s test were used as appropriate andaccepted as significant at *p* < 0.05.

## 5. Conclusions

We improved a previously developed in vitro model of cancer vs. stromal cells interaction useful for studying drug sensitivity of CCs and osteoclastogenesis without losing any information about the mutual influence of cells types. Eve was confirmed as an effective strategy to break the vicious cycle of bone metastasis. It is important to underline that this study tested Eve effect on the triple negative cell line SCP2. Since Eve plus Exemestane treatment is indicated only for metastatic ER/PGR positive BC, our results are of greater relevance as they provide the preclinical rationale to also test Eve in triple negative BC patients. Additionally, we observed a higher responsiveness to Zol of the bone metastasis model induced by triple negative BC cells. Our model seems to represent a valid platform for preclinical trials of bone-targeted drugs and for the study of the interplay of BC with bone stromal cells. Overall, these preclinical data could promote new clinical trials to provide triple negative BC patients with a valid innovative pharmacological therapy.
